# Promoting Healthy Sexual Behaviours through Comprehensive Sexuality Education

**DOI:** 10.31729/jnma.8068

**Published:** 2023-04-30

**Authors:** Rosy Shah, Amrit Pokhrel

**Affiliations:** 1 Nepalgunj Medical College, Kohalpur, Banke, Nepal

**Keywords:** *adolescent*, *medical students*, *sexual health*, *sexuality education*

## Abstract

Comprehensive sexuality education is a scientifically accurate global program that encompasses the variable aspects necessary for achieving healthy sexual and reproductive health in children of school-going age. It provides a holistic approach to developing sound knowledge and a positive attitude in a manner that does not blatantly refute the established sociocultural norms but rather delicately treads around them to bust unhealthy practices through age-appropriate measures. It is deemed necessary for health professionals to be appropriately trained to better convey sensitive information regarding sexual and reproductive well-being in a manner that is acceptable and effective, especially in the context of orthodox communities.

## INTRODUCTION

In 2018 Comprehensive sexuality education (CSE) was defined by United Nation Educational, Scientific and Cultural Organization (UNESCO).^1^ CSE is a scientifically accurate and age-appropriate global program by UNESCO that encourages health advocates to address the often overlooked and rapidly evolving concepts around sexual and reproductive health to young adults of school going age.^2^ Studies have shown that the level of existing sexuality knowledge in young people is so dire in certain areas, out of three, two girls have no contemplation of what is happening to them when they have their first menstruation.^3^ CSE addresses these key sexual and reproductive pubertal changes and incorporates associated problems like teenage pregnancy, access to contraception, trends around unsafe abortion, sexual and gender-based violence, early marriage and sexually transmitted diseases.^4^ In many Asian countries openness regarding premarital sex threatens the established societal norms and family values. The available guidelines of CSE help to explore the very sensitive aspects of sexuality without becoming flustered and without imposing ideas upon the recipient. The need for health professionals to receive formal training to be able to answer potential queries in an effective manner would help relieve the enormous health burden arising from mere ignorance.^5^

## EXPERIENCE

During our internship, we proposed to conduct a health awareness program in a local government school targeting school-going children between grades 8 and 10. At the end of our session, we hoped to better equip our target group with the basic information needed to make informed choices regarding their sexual behaviour, strengthen their relationships with their friends and family and respect and appreciate their interpersonal differences. Being medical students gave us a scientific edge in addressing the anatomical and physiological aspects of adolescence. But we understood a sensitive topic like itself would require us to be strong yet approachable to break the ice with our participants. It would be challenging to overcome reservation and shyness on both ends. There was also the risk of coming across as being "vulgar" or instigating risky behaviour by "putting ideas in one's head". We felt the need to find established and tested age-appropriate norms in communicating sexual information to the impressionable young minds of children. In doing so we discovered the CSE program by UNESCO along with several studies conceding to the efficacy of this program in perpetuating much of the same goals as ours. Opposed to the common misconception that such programs encourage young adults to engage in risky sexual acts studies showed that two in three CSE programs provided evidence of a decrease in risky sexual behaviour and encouraged responsible decision-making like the use of contraception.^6^ Being thus backed up by the scientific community we felt much more confident in putting our plans into action.

## COMPREHENSIVE SEXUALITY KNOWLEDGE IN MEDICAL STUDENTS

Before the main event, we decided to conduct a mock session amongst our own team to better apprehend the questions that could arise on an actual day. Surprisingly many of us had strikingly different views on the same topics. This instigated extensive debates and arguments among ourselves making us realise that it was necessary to recognize our goals clearly and not impose our personal beliefs or practices onto others in order to succeed. This persuaded us to seek formal training from certified CSE trainers. We felt it was important to avoid imprinting personal opinions particularly regarding grey areas into the minds of the young children and only deliver facts in the form of a clear message. Recalling our own school days brought to us the revelation that hardly any of us had any definite knowledge on a lot of sexual and reproductive topics as adolescents. Much of what young adults know is theoretical and the little practical knowledge they do have is borrowed from social platforms or from conversations with their peers. Even in medical school much of what is taught describes unhealthy sexual behaviour and hardly any literature is found painting a healthy sexual relationship. This later poses problems in the form of unclear concepts and inadequate vocabulary. Overcoming the uneasiness with our own friends and discussing openly during our training made us realize that knowing earlier what we knew then could have saved us from tremendous anxiety and uncertainty while growing up. The training proved very insightful and we realized that even after medical school there was much to learn.

## COMPREHENSIVE SEXUALITY KNOWLEDGE IN SCHOOL-GOING CHILDREN IN SUB-URBAN NEPAL

We conducted our pilot program as a tutor-based peer-to-peer learning with two tutors, a male and a female, assigned to a total of 20 trainees. It comprised health education, demonstration, information exchange and active question-and-answer sessions. Young adults are naturally inquisitive and have the ability to soak up information like a sponge. But even so, as anticipated talking about anything even remotely associated with sex caused them to initially show inhibition and shyness. But once they overcame the awkwardness we found them to be remarkably inquisitive and brimming with queries. The open and casual discussion enabled us to unmask traditional firmly rooted beliefs like menstruation being a time of "ridding body of impure blood" and the lack of clarity regarding bodily processes like "menstrual blood flows from the same opening as that for urine". Busting myths in a delicate yet affirmative manner was the most challenging of all tasks but our formal CSE training had prepared us for that part. Above everything we had learnt that it was necessary to respect our subjects for them to reiterate our teachings. In several instances, we felt that the training had prepared us to avoid potential controversies and grey moral grounds while still providing an appropriate and acceptable response. The receptiveness and kindness that the children demonstrated were a fresh breath of air. We were truly overwhelmed with how fulfilled we felt towards the end of the program and this has only fuelled us to conduct more such programs thereafter. We understand that a limitation to CSE programs, especially in South Asian settings due to cultural normative patterns can be the elimination of dialogue regarding pleasure and sexual fluidity. This may be due to the portrayal of sex as problematic behaviour and not as a natural entity.^7^

## WAY FORWARD

Available literature from the last three decades offers strong evidence for the effectiveness of CSE. School programs can be framed to be initiated early on and delivered successively over increasing grades to build up knowledge in a manner that is age appropriate and essential. Health professionals should seek to be better informed regarding the aspects of CSE to delve better into prevention medicine. The need for formal training on CSE is necessary to better reiterate learning objectives without potentially harming the existing cultural sentiments and dissuading the participants. It would also help in acquiring clear concepts and an appropriate vocabulary to interact without becoming flustered while remaining respectful to the target audience. As medical professionals, we are capable of sending out enormous ripples of positive changes. Only through formal training and indulgence in research can we equip ourselves for the battle against ignorance. It is upon ourselves to seek to become adequate before we begin to address inadequacies. While there may be limitations to what can be comfortably included in our discussions, it is time we start where we stand and begin the climb to a better view.
